# Key Macrophage Responses to Infection With *Mycobacterium tuberculosis* Are Co-Regulated by microRNAs and DNA Methylation

**DOI:** 10.3389/fimmu.2021.685237

**Published:** 2021-06-01

**Authors:** Monika Looney, Rachel Lorenc, Marc K. Halushka, Petros C. Karakousis

**Affiliations:** ^1^ Department of Medicine, Division of Infectious Disease, Johns Hopkins University School of Medicine, Baltimore, MD, United States; ^2^ Department of Pathology, Johns Hopkins University School of Medicine, Baltimore, MD, United States

**Keywords:** tuberculosis, macrophages, microRNAs, methylation, host response

## Abstract

Tuberculosis (TB) is the leading cause of death from infection with a single bacterial pathogen. Host macrophages are the primary cell type infected with *Mycobacterium tuberculosis* (*Mtb*), the organism that causes TB. Macrophage response pathways are regulated by various factors, including microRNAs (miRNAs) and epigenetic changes that can shape the outcome of infection. Although dysregulation of both miRNAs and DNA methylation have been studied in the context *of Mtb* infection, studies have not yet investigated how these two processes may jointly co-regulate critical anti-TB pathways in primary human macrophages. In the current study, we integrated genome-wide analyses of miRNA abundance and DNA methylation status with mRNA transcriptomics in *Mtb*-infected primary human macrophages to decipher which macrophage functions may be subject to control by these two types of regulation. Using *in vitro* macrophage infection models and next generation sequencing, we found that miRNAs and methylation changes co-regulate important macrophage response processes, including immune cell activation, macrophage metabolism, and AMPK pathway signaling.

## Introduction

Tuberculosis (TB) is one of the top 10 causes of death worldwide and the leading cause of death from a single bacterial pathogen (1). It is estimated that approximately one quarter of the world is currently infected with *Mycobacterium tuberculosis* (*Mtb*), the bacterium that causes TB.

Macrophages are the primary cell type infected by *Mtb.* The host’s ability to control *Mtb* infection is dependent upon regulation of various cellular processes, including activation of macrophages to promote killing of intracellular *Mtb*, and cell-to-cell signaling to coordinate innate and adaptive immune responses (2–5). However, *Mtb* has evolved virulence mechanisms to suppress host defenses and promote its own survival within the host (3, 6). While it is known that *Mtb* infection drives changes in expression of host genes involved in these pathways, mechanisms by which *Mtb* alters host transcriptional responses to subvert macrophage-mediated killing are not well understood.

Many key host transcriptional pathways are controlled by microRNAs (miRNAs) and epigenetic changes (primarily methylation of promoter regions). *Mtb* infection has been shown to dysregulate miRNA expression and alter DNA methylation patterns in infected host cells (7, 8). However, most of the published studies in this area suffer from two critical limitations: 1) biased or limited scope of analysis, and 2) reliance on cancerous cell lines (9–14). Previous studies on miRNA and epigenetic regulation focus primarily on specific targets of interest based on their known function in TB disease (9–13). Although targeted approaches are very powerful for examining the role of specific miRNAs and methylation changes in *Mtb-*host interactions, they are unable to provide a global understanding of transcriptional networks regulating host defenses against *Mtb* infection and cannot identify novel host regulatory factors involved in these processes. Furthermore, transcriptional and epigenetic analysis of immortalized cell lines is limited by the observation that such cells at baseline display dysregulation of small regulatory RNAs, epigenetic markers, and messenger RNA (mRNA) (9, 14). Therefore, while these models have facilitated major research developments for understanding macrophage responses to *Mtb*, it is important that the regulatory functions of miRNAs and DNA methylation also be studied in primary human macrophages.

We hypothesized that *Mtb* alters transcriptional responses in infected macrophages to favor intracellular bacillary survival by modulating the expression of key miRNAs and the methylation of important host defense genes. Using unbiased next-generation sequencing (NGS) and high-throughput DNA methylation profiling, we developed an integrated analysis of dysregulated small RNAs, methylation, and transcriptional pathways in *Mtb* infection of human monocyte-derived macrophages (MDMs).

## Materials and Methods

### Ethics

The study was reviewed by the Johns Hopkins University Institutional Review Board and it was determined that it does not constitute human subjects research under the DHHS or FDA regulations. The authors did not have any contact with donors. All samples were de-identified by the Blood Donor Center of the Anne Arundel Medical Center, Maryland, USA prior to use in these studies.

### Bacterial Cultures

The virulent *Mtb* strain H37Rv-*lux* was used for all studies (15). H37Rv-*lux* contains the full bacterial luciferase operon, *luxAB*, which constitutively expresses luciferase and its substrate, luciferin. A robust luminescent signal is produced, which can be measured in relative light units (RLU) and which serves as a reliable and instantaneous readout for colony forming units (CFU). H37Rv-*lux* was cultured in 7H9 + 10% OADC + 0.05% Tween-80 + 0.2% glycerol at 37°C in a shaking incubator or made into frozen stocks kept at -80°C in 7H9 + 10% OADC + 0.05% Tween-80 + 10% glycerol. Three frozen stocks were thawed and used to confirm a viable bacterial density of 1x10^8^ CFU/ml. These frozen stocks were used directly for infection of primary human MDMs.

### Isolation of Primary Human MDMs

Primary human peripheral blood mononuclear cells (PBMCs) were isolated from platelet-depleted whole blood from healthy human donors using standard Ficoll-paque density gradient centrifugation (GE Healthcare, Cat# 17144003). Both male and female donors were used (donors A and D were male, donors B and C were female). Monocytes were isolated from the buffy coat using passive plastic adherence to cell culture plates at 37°C, 5% CO_2_, for 4 hours in serum-free media (RPMI-1640 + 4mM L-glutamine). After a 4-hour incubation, non-adherent lymphocytes and erythrocytes were removed with five washes in 1X phosphate buffered saline (PBS). Adherent monocytes were allowed to differentiate into macrophages over a period of one week at 37°C, 5% CO_2_, in complete media containing 10% non-heat inactivated fetal bovine serum (FBS) (RPMI 1640 + 4mM L-glutamine + 10% FBS). Media was changed every 2-3 days. On day 7, macrophages were infected with *Mtb*. Colony-stimulating factors (i.e. M-CSF and GM-CSF) were intentionally excluded from culture media as 10% FBS alone allows for differentiation of primary monocytes into macrophages (16) and to maintain natural cell heterogeneity and avoid artificial primining of macrophages to develop M1 or M2 phenotypes (17).

### Infection of MDMs With *Mtb*

After 7 days of differentiation, primary human MDMs were infected with *Mtb* H37Rv-*lux* at a multiplicity of infection (MOI) of 5 or 10 for 24 or 48 hours. Infected cells and uninfected controls were incubated in complete cell culture media (described above) at 37°C, 5% CO_2_. At each time point post-infection, MDM viability was measured by 3-(4,5-dimethylthiazol-2-yl)-5-(3-carboxymethoxyphenyl)-2-(4-sulfophenyl)-2H-tetrazolium) (MTS) assay (Promega, CellTiter 96^®^ AQueous One Solution Cell Proliferation Assay, Cat # G3582). After recording MDM viability, MDMs were lysed in 0.05% sodium dodecyl sulfate (SDS) and measured for bacterial burden by single tube luminometer (**Supplemental Figure 1**, data previously shown) (18). Matched wells for each sample were also harvested for RNA isolation in TRIzol (ThermoFisher).

### Small RNA Isolation, Library Preparation, Sequencing, and Analysis

Total RNA, including small RNA, was isolated from samples frozen at –80°C in TRIzol using the Qiagen miRNeasy mini kit (Qiagen, Cat#217004). Manufacturer instructions for isolation were followed exactly. Quality of total and small RNA were assessed using a fragment analyzer at the Johns Hopkins University DNA Services Core Facility.

RNA was converted into small RNA libraries for small RNA sequencing (sRNA-seq) using the Qiagen QIAseq miRNA Library Kit (Qiagen, Cat# 331505). Quality, size, and concentration of small RNA libraries were then assessed using a fragment analyzer at the Johns Hopkins University DNA Services Core Facility. Libraries were pooled to 1ng/μl and sequenced in a single run on a NextSeq 500 instrument to a depth of at least 1 million reads per sample at the Johns Hopkins University Transcriptomics and Deep Sequencing Core Facility (JHU TSC).

sRNA-seq raw data were aligned using miRge2.0, a bioinformatic sequencing analysis tool designed specifically for processing sRNA-seq data (19). Differential expression of miRNAs was compared using the DESeq2 package, including Benjamini Hochberg correction, in R. miRNA target analysis was performed using miRNet2.0 (20). Pathway enrichment analysis of differently expressed miRNA targets was generated using the R package gprofiler2 (21).

### Total RNA Isolation, Library Preparation, Sequencing, and Analysis

Total RNA from MDMs infected at an MOI of 10 for 48 hours was isolated with the Qiagen AllPrep DNA/RNA Mini Kit (Qiagen, Cat # 80204) following manufacturer instructions. Quality of RNA was assessed using a fragment analyzer at the JHU TSC.

Purified total RNA was submitted to the JHU TSC for library preparation and sequencing. RNA was converted into total RNA libraries using Illumina TruSeq Stranded Total RNA Library Prep Kit (Illumina, Cat # 20020597). RNA library quality, size, and concentration were assessed using a fragment analyzer at the JHU TSC. Libraries were pooled to 2nM and sequenced in a single run on a NextSeq 500 instrument by single-end sequencing to a depth of approximately 50 million reads per sample with a read length of 75 base pairs. Reads were aligned using the Hisat2, Stringtie, Ballgown pipeline described previously (22). Differential expression was assessed using the DESeq2 package in R. Benjamini Hochberg correction was used.

### DNA Isolation, Whole Genome Bisulfite Sequencing Library Prep, Sequencing, and Analysis

Genomic DNA from MDMs infected at an MOI of 10 for 48 hours was isolated in parallel with total RNA from identical samples using the Qiagen AllPrep DNA/RNA Mini Kit (Qiagen, Cat# 80204). Purified DNA was submitted to the JHU TSC and sent to Novogene Co., Ltd. for quality control check, bisulfite conversion, library preparation, whole genome bisulfite sequencing (WGBS), and preliminary analysis. DNA was fragmented with a Covaris S220 to generate fragments of 200-300bp in length. Adapter ligation and library preparation was performed using the EZ DNA Methylation Gold Kit (Zymo Research, Cat # D5005). Quality control was performed using a Qubit2.0 and Aligent 2100 bioanalyzer and samples were pooled to a concentration of 2nM. Libraries were sequenced on an Illumina HiSeq platform using paired-end sequencing. The data analysis pipeline involved alignment to the Ensemble *Homo sapiens* reference genome version GRCH83 release 92 and quantification using Bismark (23). Analysis of differentially methylated regions (DMRs) was performed using DSS (24–26). Gene ontology enrichment analysis of DMRs was done using GOseq (27). Integration with sRNA-seq and RNA-seq was performed in R.

## Results

### miRNAs in Primary Human MDMs Are Dysregulated by *Mtb* Infection

To investigate *Mtb*-driven changes in miRNA expression of primary human macrophages, we isolated MDMs from healthy donors and infected them *ex vivo* with *Mtb* strain H37Rv-*lux*, which produces a bioluminescent signal that serves as a real-time immediate measure of bacterial burden (15). At each time point (24 or 48 hours post-infection (h.p.i.)), cells were assessed for viability and bacterial burden. Through 48 hours of infection, there were no significant changes in MDM viability whereas *Mtb* burden increased with both time post-infection and MOI, suggestive of a productive infection (data previously shown) (18). RNA from matched wells was harvested for library prep and sRNA-seq (**Supplemental Figure 1**).

**Figure 1:**
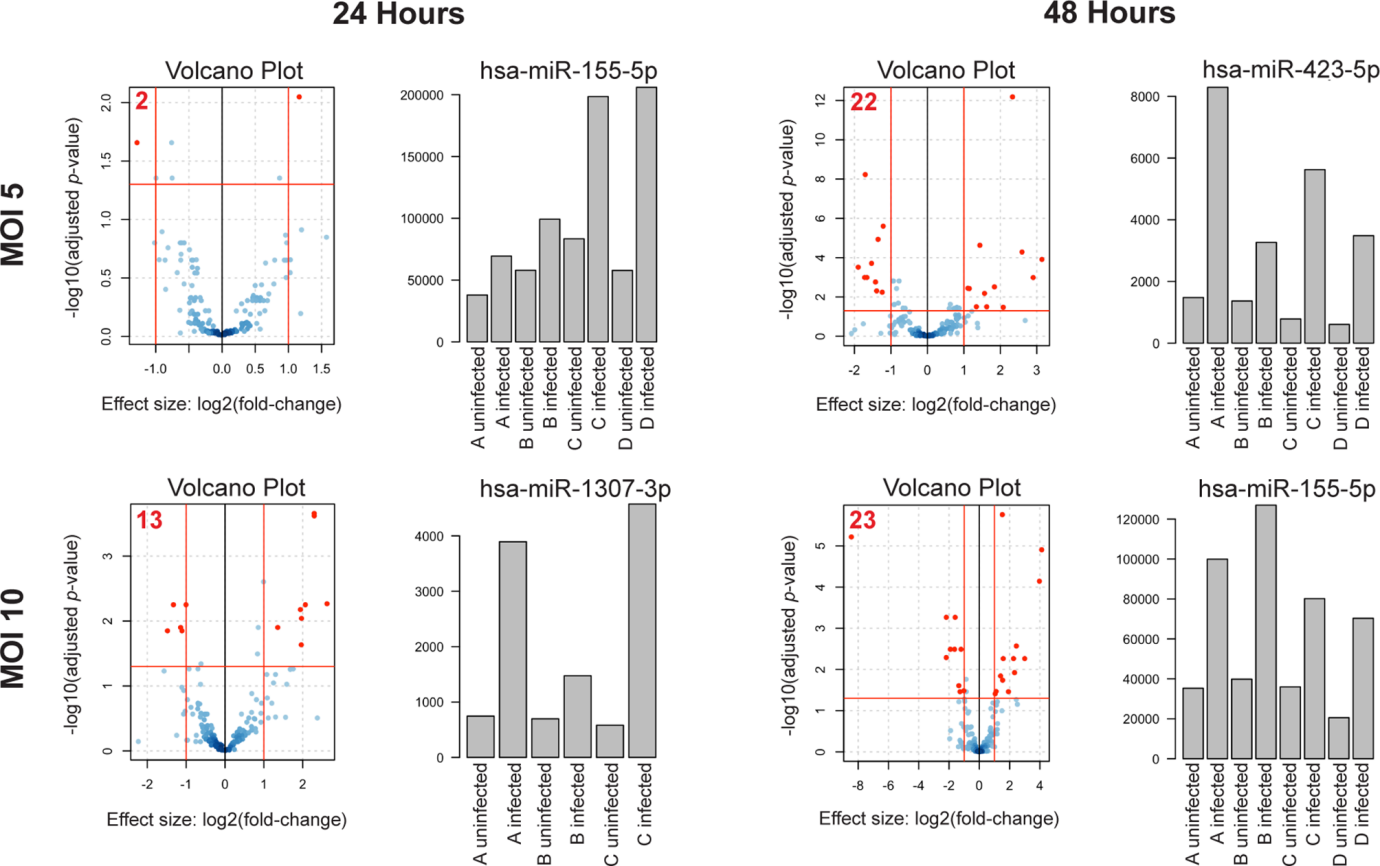
miRNA dysregulation increases with time post-infection and MOI. Volcano plots of differentially expressed miRNAs in each condition. n = 4 independent human donors. Red lines represent significance thresholds, red numbers represent total number of significantly DE miRNAs. Bar plots to the right of each volcano plot show the most significantly dysregulated miRNA for that condition. Bar plot y-axis shows normalized read counts.

Consistent with the published literature in cell lines (14), we found that some miRNAs in primary human MDMs were significantly dysregulated following infection with virulent *Mtb* (**Figure 1** and **Supplemental Table 1**). Additionally, we found that the number of dysregulated miRNAs and the magnitude of dysregulation increased both with time post-infection and MOI, suggesting that miRNA dysregulation increases with severity of infection. In the mildest infection condition (MOI= 5 at 24 h.p.i.), there were only two miRNAs which were significantly dysregulated. In contrast, 22 miRNAs were significantly differentially regulated at 48 h.p.i. At MOI= 10, the number of dysregulated miRNAs increased from 13 at 24 hours to 23 at 48 h.p.i. (**Figure 1**, Volcano plots and **Table 1**). We did not find any differences in miRNA expression in uninfected MDM controls between the 24 and 48 hour time points, suggesting there is little baseline variability in uninfected controls. As expected, considering all infection conditions together, we found that some miRNAs were significantly differently expressed at 48 hours compared to 24 hours (**Supplemental Figure 2**).

**Table 1:** Significantly downregulated and upregulated miRNAs for each condition.

MOI	Time Post Infection	Downregulated miRNAs	Upregulated miRNAs
5	24 hours	miR-340-5p	miR-155-5p
5	48 hours	miR-340-5p miR-374a-5p miR-30b-5p/30c-5p miR-22-5p miR-374b-5p miR-22-3p miR-15a-5p miR-660-5p miR-365a-3p/365b-3p miR-21-5p	let-7a-5p/7c-5p miR-1307-3p miR-3615-3p miR-155-5p miR-148a-3p miR-191-3p miR-486-5p miR-328-3p miR-423-5p miR-1275 miR-122-5p miR-12136
10	24 hours	miR-19b-3p miR-374a-5p miR-15a-5p miR-30b-5p/30c-5p miR-34a-5p	miR-423-3p miR-486-5p miR-328-3p miR-3615-3p miR-423-5p miR-1307-3p miR-501-3p miR-12136
10	48 hours	miR-1307-5p miR-374a-5p miR-340-5p miR-365a-3p/365b-3p miR-22-3p miR-30b-5p/30c-5p miR-374b-5p miR-21-5p miR-185-5p miR-23a-3p/23b-3p	let7b-5p miR-132-5p miR-148a-3p miR-155-5p miR-501-3p miR-1307-3p miR-664a-5p miR-423-5p miR-191-3p miR-486-5p miR-1275 miR-122-5p miR-12136

Next, we examined which miRNAs were most significantly dysregulated in each infection condition. miR-155-5p was consistently and significantly upregulated in both the least (MOI of 5, 24 h.p.i.) and most severe (MOI of 10, 48 h.p.i.) conditions (**Figure 1**, Bar plots). miR-155-5p was also significantly upregulated at MOI= 5/48 h.p.i., although not as significantly as miR-423-5p. miR-1307-3p, the most significantly dysregulated miRNA at MOI= 10/24 h.p.i. was also upregulated, although it was not significantly dysregulated in other conditions.

Taken together, these results suggest that the degree of miRNA dysregulation is driven by severity of *Mtb* infection and that miRNAs which are dysregulated in more than one condition, such as miR-155-5p, may be important regulators of the macrophage response to *Mtb*.

### Bioinformatic Analysis and Literature Review Identified 10 Candidate miRNAs for Further Analysis

**Figure 2:**
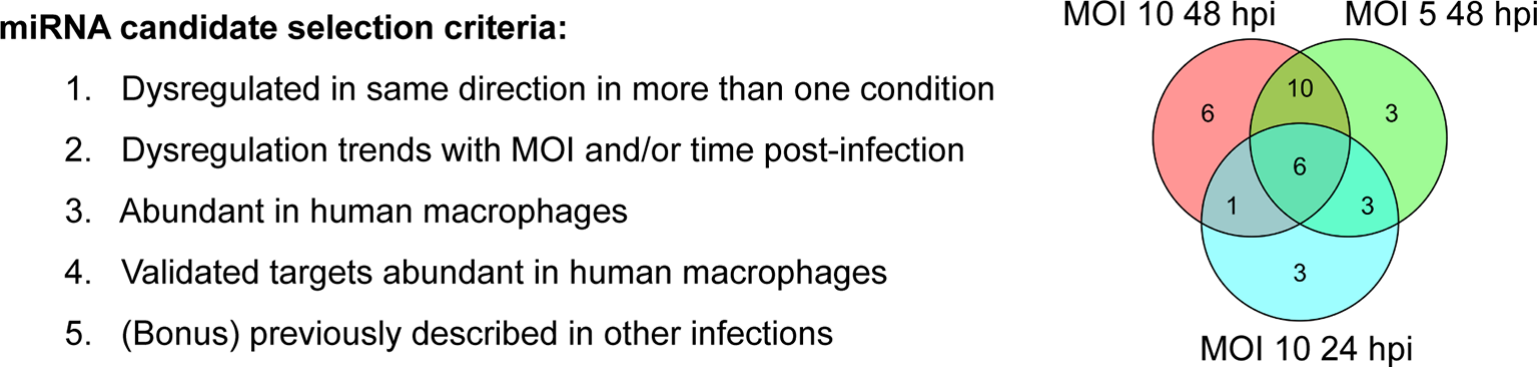
Selection of miRNA candidates for pathway analysis. NGS analysis and literature review led to selection of 10 candidate miRNAs and their respective targets. Left) Selection criteria for candidate miRNAs. Right) Venn Diagram showing 20 differentially expressed miRNAs shared between the three conditions in which we observed the most significant miRNA dysregulation (top 3).

In order to gain a deeper understanding of how dysregulated miRNAs may be altering the macrophage response to infection with *Mtb*, we developed the following set of criteria for prioritizing miRNAs for further study: 1) dysregulation of the miRNA must be significant in more than one of the four infection conditions, and must be consistently dysregulated in the same direction (i.e., consistently up or down regulated); 2) dysregulation of the miRNA must trend with MOI or time post-infection; 3) the miRNA itself must be known to be abundant in human macrophages (as determined by MiRgeneDB (28); 4) known, experimentally validated, targets of the miRNA must also be abundant in human macrophages (as determined by The Human Protein Atlas (29) to ensure stoichiometric probability of miRNA-target interaction in our cell type of interest); and 5) miRNAs which have been previously described as being involved in other forms of infectious cell stress are given special consideration as additional positive controls (**Figure 2**).

From our own sRNA-seq dataset, we identified 6 significantly dysregulated miRNAs fitting our selection criteria. These miRNAs were miR-155-5p, miR-191-3p, miR-22-3p, miR-21-5p, miR-30b-5p, and miR-30c-5p. Due to their high degree of sequence similarity, miR-30b-5p and miR-30c-5p are combined for sequencing alignment in miRge2.0. However, as they have some distinct gene targets, for integrated network analysis, miR-30b-5p and miR-30c-5p were separated and analyzed as distinct entities. We added 4 additional literature-identified candidate miRNAs based on criterion #5, which we included given that they are known to be involved in altering cellular responses during infection: miR-223-5 (10), miR-29a-3p (9), miR-27a-3p (11), miR-125b-5p (12). These additional four candidate miRNAs were each dysregulated in a consistent direction in more than one condition, but did not meet our conservative thresholds for significance (log_2_(fold-change) > 1; adjusted p-value < 0.01). These 10 miRNAs became candidates for further functional pathway analysis (**Table 2**).

**Table 2:** List of candidate miRNAs to be used for further analysis.

Source	miRNA
NGS	miR-155-5pmiR-30b-5p miR-30c-5p miR-191-3p miR-22-3p miR-21-5p
(9)	miR-29a-3p
(10)	miR-223-5p
(11)	miR-27a-3p
(12)	miR-125b-5p

Final selection of miRNA candidates for ongoing mechanistic analyses. 6 miRNAs that met selection criteria were selected from 20 miRNAs differentially expressed in more than one “top 3” condition. 4 additional literature-identified candidates were selected based on their representation in previously published relevant studies.

### Many mRNAs Dysregulated in *Mtb*-Infected MDMs Are Targets of Candidate miRNAs

**Figure 3:**
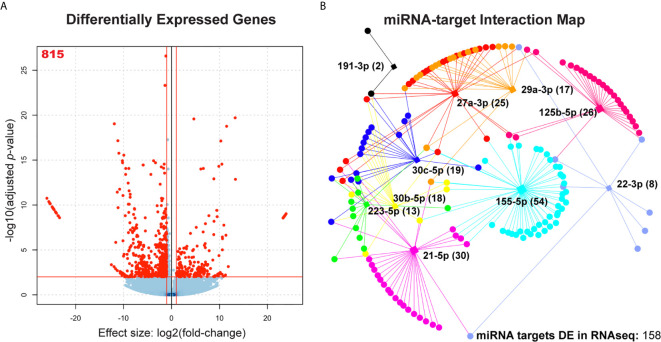
Association of significantly dysregulated miRNAs with differentially expressed cognate mRNAs during *Mtb* infection of human MDMs. **(A)** Volcano plot showing all differentially expressed mRNAs during *Mtb* infection. n = 4 independent human donors. Red lines represent significance thresholds, red number represent total number of significantly DE mRNAs. **(B)** Interaction map showing network of candidate miRNAs and their respective targets. Square nodes represent each candidate miRNA (labeled). Circular nodes and points represent differentially expressed gene targets (targets identified by miRTarBase v8.0 and miRNet). Numbers in parentheses beside each miRNA label represent the number of gene targets that are also significantly dysregulated in the RNAseq results. Each miRNA node is uniquely color-coded. Edges that extend between miRNA nodes and gene and gene target nodes are coded to the same color of their respective miRNA regulator.

To assess dysregulation of host cell pathways at the mRNA level, we performed RNA-seq of total RNA extracted from uninfected MDMs and MDMs infected at an MOI of 10 for 48 hours. A total of 815 mRNAs were significantly dysregulated in *Mtb* infection (log_2_(fold-change) > 1; adjusted p-value < 0.05) (**Figure 3A** and **Supplemental Table 2**). Gene Ontology Biological Process (GO : BP) functional enrichment analysis showed that, as expected, the most significantly dysregulated processes associated with the 815 differentially expressed genes included leukocyte activation, coordination of immune signaling and response cascades, exocytosis, apoptosis, and fatty acid and lipid metabolism (**Supplemental Table 3**). Each of these functions play key roles in clearance of *Mtb* infection (3, 4, 6, 30–32). We compared these significantly dysregulated genes to a comprehensive list of all 3044 known targets of the 10 candidate miRNAs (via miRTarBase v8.0) (20) to generate a profile of 158 genes which are both targeted by at least one candidate miRNA and significantly dysregulated in our RNA-seq dataset (**Figure 3B**). The number of dysregulated genes that were also targets of at least one candidate miRNA ranged between miRNA candidates. For instance, miR-155-5p had the most, with 54 target genes that were dysregulated during *Mtb* infection, while miR-191-3p only had two (**Figure 3B**). All miRNA candidates had at least two significantly dysregulated gene targets.

We found that 119 out of the 158 dysregulated target genes were downregulated (75.3%), however, each miRNA had both down and upregulated targets in *Mtb* infection, suggesting direct and indirect regulation (**Figure 3B**). It is likely that those genes which are dysregulated in the opposite direction of their associated miRNA are more likely to be targeted by that miRNA during *Mtb* infection (33). To determine if the genes dysregulated in our RNA-seq dataset were enriched for candidate miRNA targets, we performed a Chi-square analysis with Yates correction using a two by two contingency table. We found that the set of 815 differentially expressed genes is enriched for targets of our 10 miRNAs of interest (X^2^ = 14.86, z = 3.86, *p* = 0.0001), suggesting that it is unlikely that dysregulation of miRNA candidate genes would occur solely due to random chance (**Supplemental Table 4**). Odds ratio calculation shows that it is 1.42 times more likely for dysregulated mRNAs to be candidate miRNA targets compared to non-targets.

### Functional Enrichment Analysis for Significantly Dysregulated miRNA Targets

**Figure 4:**
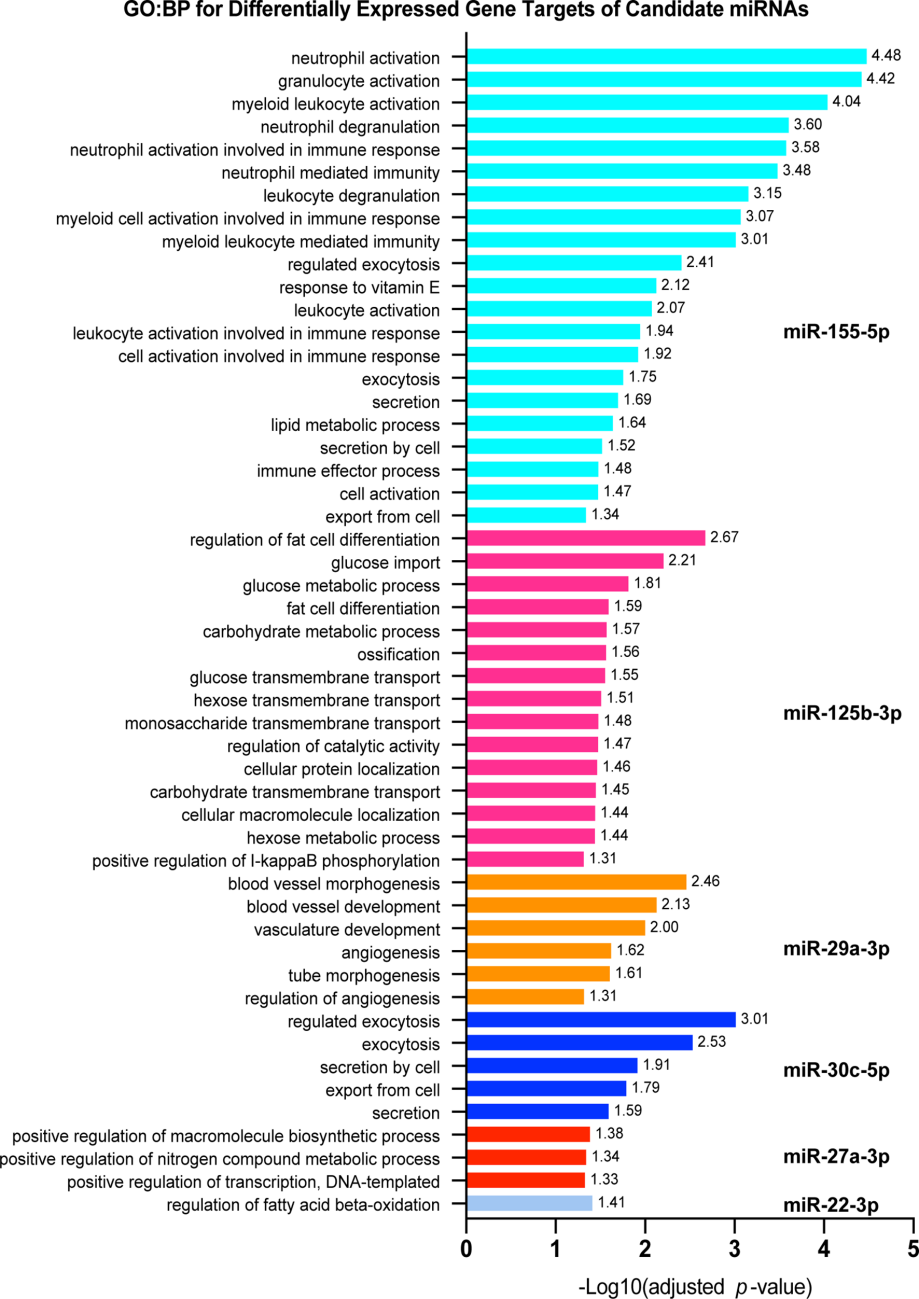
Pathway analysis for significantly dysregulated targets of candidate miRNAs. Each shows gprofiler2 (g:OSt) results for a miRNA candidate. g:OSt was used to analyze the association of each miRNAs target genes with Gene Ontology biological processes functional groups (GO : BP). All significant GO : BP associations are reported. miRNAs which do not have GO : BP results listed did not have significant associations with any GO : BP functional group. g:OSt MIRNA was used to determine which miRNA is most significantly associated with each list of dysregulated targets. The top miRNA hit for each is reported.

To investigate which host cell response pathways are associated with our observed networks of miRNA-target regulation, we used the set of genes targeted by each candidate miRNA, independently, and performed functional enrichment analysis and miRNA association validation. Using gprofiler2 in R, we searched for GO : BP that were significantly associated with the dysregulated target genes for each candidate miRNA. Six candidate miRNAs were associated with biological processes critical for defense against *Mtb* infection (**Figure 4**). Specifically, 18 significantly dysregulated genes targeted by miR-155-5p were associated with activation of various immune cells (primarily those in myeloid cell lineages), 16 were associated with exocytosis and secretion, and 15 were involved in regulation of lipid metabolism. miR-125b-5p, miR-27a-3p, and miR-22-3p targets were primarily involved in regulation of metabolism and lipid processing, where 23 differentially expressed gene targets were involved in these processes. Specific processes included glucose metabolism, macromolecule biosynthesis, and fatty acid oxidation. miR-125b-5p also had 11 differentially expressed targets involved in protein and small molecule transport and subcellular localization. Nine targets of miR-30c-5p were involved in regulation of exocytosis and secretion and 7 targets of miR-29a-3p were associated with regulation of blood vessel development and regulation of angiogenesis. The significantly dysregulated target genes for miR-223-5p, miR-191-3p, miR-30b-5p, and miR-21-5p did not significantly associate with any particular GO : BP functional group. Each list of target genes was most significantly associated with the miRNA regulator we had matched it to in our network analysis, giving further support to the relationship between each miRNA and its corresponding target list.

Though these results show that many differentially expressed targets of our 10 candidate miRNAs are involved in regulation of key macrophage processes relevant to the defense against *Mtb* infection, it is likely that they are also regulated by other factors, such as DNA methylation.

### Expression of Immune Cell Pathway Genes Is Also Influenced by Remodeling of Methylation

A critical mechanism for pre-transcriptional regulation of immune cell function involves alternation of methylation patterns that may increase or decrease transcriptional machinery access to different gene regions (34). Evidence suggests that *Mtb* alters epigenetic markers, such as methylation of promoter regions, to alter gene expression in host cells (35). However, these methylation changes have not been assessed at a genome-wide scale in *Mtb*-infected primary macrophages. Furthermore, miRNA expression may also be affected by epigenetic changes and it remains to be determined how miRNAs and methylation may work together to co-regulate important macrophage signaling pathways. Therefore, we hypothesized that *Mtb* reprograms the macrophage response by integrating pre-transcriptional regulation *via* methylation and post-transcriptional regulation *via* miRNAs of the same pathways. We also posited that changes in candidate miRNA expression may be due to differences in methylation of miRNA promoter regions. To test this, we performed whole genome next-generation bisulfite sequencing (WGBS) on the same *Mtb*-infected primary human MDMs used for RNA-seq analysis (**Figure 3**). We then analyzed our WGBS, RNA-seq and sRNA-seq datasets to identify intersecting networks between these regulatory systems.

**Figure 5:**
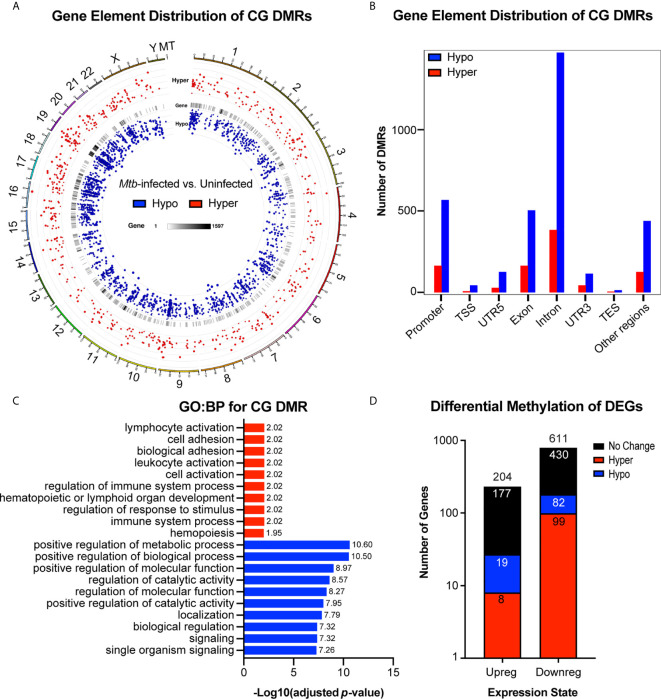
Genome wide differential CG methylation patterns show enrichment for genes associated with immune activation and metabolic processing. **(A)** Genome wide chromosomal alignment of hypo (blue) and hyper (red) methylated CG sites in *Mtb*-infected MDMs. **(B)** Distribution of hypo and hypermethylated CGs (DMRs) over each genetic element. **(C)** Functional enrichment gene ontology biological process (GO : BP) analysis for all differentially methylated CGs. -Log_10_(adjusted *p*-value) values are reported to the right of each bar. GO : BP terms associated with hypermethylated CG DMRs are highlighted in red. Terms associated with hypomethylated CG DMRs are highlighted in blue. **(D)** Total number of up and downregulated genes from RNA sequencing results (**Figure 6**) with overlay of genes that are also hypo or hypermethylated. Number in each color coded segment represents total number of genes with a specific expression and methylation state. Number above each bar represents total up or downregulated genes. *n*=4. MDMs were infected with *Mtb* H37Rv for 48 hours at MOI= 10.

We found that differential methylation of CG sites (DMRs, length > 50bp, target site inside DMR ≥ 3CG, *p* < 1x10^-5^) were evenly distributed across chromosomes and that more DMRs were hypomethylated rather than hypermethylated (**Figures 5A, B** and **Supplemental Table 5**). We also found that differential methylation patterns were most common within introns, followed by promoter regions and exons (**Figure 5B**). A growing body of evidence suggests that methylation changes in non-promoter regions are relevant to epigenetic regulation of gene expression. Previously, methylation of introns was thought to serve little function, but has recently been shown to exhibit an inverse relationship with gene expression and may be implicated in progression of cancer (36, 37). Functional enrichment GO : BP analysis revealed that hypermethylated CG DMRs were most associated with pathways involved in immune cell activation, while hypomethylated DMRs were most associated with alteration of positive regulation of metabolic processes (**Figure 5C**). We then compared the set of genes we found to have differentially methylated CG regions to the differentially expressed genes identified by our RNA-seq study (**Figures 3**, **5D**). Importantly, among the upregulated genes, there were more hypomethylated genes (19) compared to hypermethylated genes (8). Similarly, we found that more downregulated genes were hypermethylated (99) rather than hypomethylated (82) (**Figure 5D**). Chi-square analysis revealed that upregulated genes were not statistically enriched for hypomethylated DMRs (*p* = 0.08) and that downregulated genes were significantly less likely to be hypermethylated compared to no methylation change (*p* = 0.0002), suggesting that differential methylation alone is not sufficient to explain differences in mRNA expression, and that other regulatory systems, such as miRNAs, are likely to contribute to overall changes in gene expression. Differential methylation of candidate miRNA-associated gene regions was found for miR-125b, though the differences in methylation were not significant, suggesting that miRNAs and methylation changes are acting largely independently in regulating mRNA expression.

**Figure 6:**
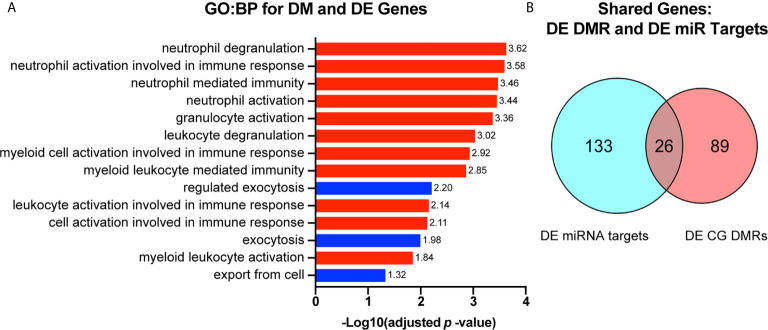
Genes that are both differentially methylated and differentially expressed are involved in immune cell functioning and are subject to regulation by candidate miRNAs. **(A)** Genes that were both differentially methylated and differentially expressed were selected and used for gene ontology biological process (GO : BP) functional enrichment analysis. -Log_10_ (adjusted *p*-value) value for each GO : BP term is reported to the right of each bar. GO : BP terms related to immune cell activation are highlighted in red. Those related to exocytosis and secretion are highlighted in blue. **(B)** Genes that were both differentially methylated and differentially expressed were compared to genes that we found to be differentially expressed and targeted by at least one candidate miRNA. Overlap represents the number of genes that are differentially expressed by RNAseq, targets of one or more miRNA candidate, and differentially methylated.

To select only genes which may be directly influenced by changes in DNA methylation, we identified genes that were both differentially expressed and divergently methylated. We next sought to determine which macrophage functions may be influenced by alterations in expression of genes with differential methylation. We found that differentially methylated genes that were also differentially expressed in either direction were associated with regulation of immune cell activation and exocytosis (**Figure 6A**). We then sought to determine if any genes that were divergently methylated and differentially expressed were also targets of any of the previously identified candidate miRNAs. We found 26 genes that were divergently methylated, differentially expressed, and targeted by at least one candidate miRNA (**Figure 6B**).

**Figure 7:**
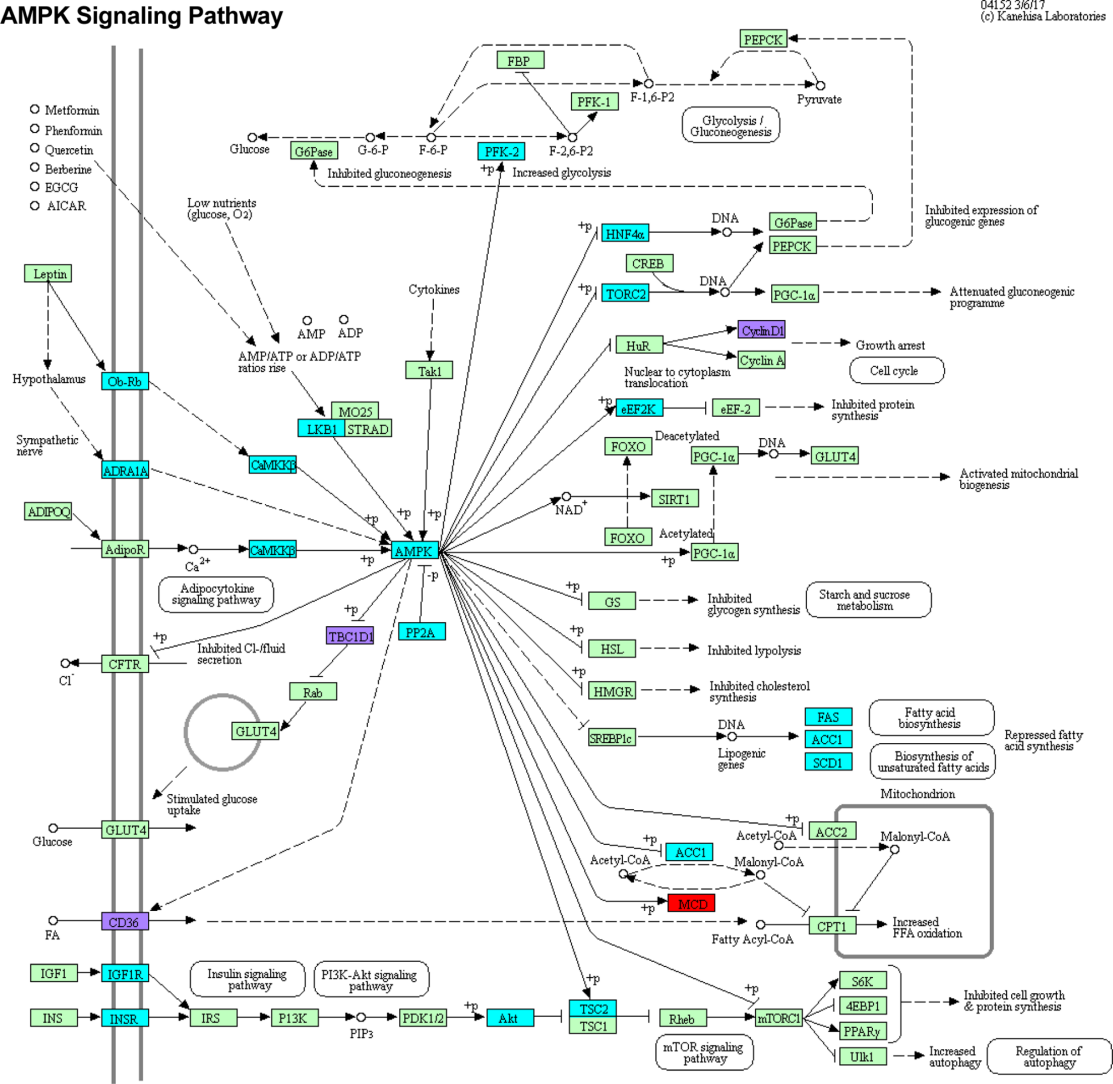
DMRs DEGs and DE miRNA targets intersect at the AMPK signaling pathway. Genes that were differentially methylated, differentially expressed, or targeted by candidate miRNAs were subjected to functional enrichment KEGG analysis. These genes were most significantly associated with the AMPK signaling pathway. Genes which are only differentially methylated are highlighted in cyan. Genes which are only differentially expressed are highlighted in red. Genes which are differentially methylated, differentially expressed, and regulated by one or more miRNA are highlighted in purple. Light green boxes represent other pathway genes that were not found to be significantly in our studies.

Functional enrichment KEGG analysis of this set of 26 genes revealed a tight association with the AMPK signaling pathway (**Figure 7**). Three of these 26 genes, CyclinD1 (CCND1, an important regulator of cell proliferation), TBC Domain Family Member 1 (TBC1D1, regulator of cell growth and differentiation), and cluster of differentiation 36 (CD36, involved in antigen processing, cross presentation, and low density lipoprotein binding), are central to the AMPK pathway. CD36 is a target of miR-155-5p, TBC1D1 is a target of miR-125b-5p, and CCND1 is a target of miR-155-5p, miR-223-5p, miR-27a-3p, and miR-29a-3p (38). The AMPK pathway also contains various genes that were differentially methylated alone, and one gene, C-C Motif Chemokine Ligand 22 (MCD, a chemoattractant for various immune cells), that was differentially expressed despite lack of differential methylation or targeting by any candidate miRNA. Chi-square analysis shows that the dysregulation of the AMPK pathway is statistically significant (X^2^ = 22.43, z = 4.74, *p* = <0.0001) and unlikely to be due to random chance (**Supplemental Table 6**). An odds ratio of 3.30 suggests that dysregulated genes are over three times more likely to be involved in the AMPK pathway than unrelated pathways.

## Discussion

Taken together, our results show that critical innate immune processes and signaling are influenced by both pre-transcriptional regulation *via* changes in DNA methylation and post-transcriptional regulation *via* altered miRNA expression. Application of next generation sRNA-seq allowed us to identify a small profile of candidate miRNAs that are likely to serve a biological function during *Mtb* infection. By integrating our sRNA-seq data with total RNA-seq, we found that all of our candidate miRNAs have mRNA targets that were also differentially expressed in *Mtb*-infected cells. These miRNA-targeted differentially expressed genes are involved in various biological processes that are critical for the host defense against *Mtb* infection. The pathways that are most significantly represented amongst these miRNA-targeted differentially expressed mRNAs included innate immune cell activation, regulation of metabolic and lipid synthesis processes, vasculature development, and intracellular transport, exocytosis, and secretion.

Though this study is the first to integrate multiple next-generation sequencing-based analyses of miRNAs, mRNAs, and methylation in primary human MDMs infected with *Mtb*, previous studies have investigated each of these components individually and in different models. Consistent with the published literature, we found that miR-155-5p was robustly upregulated. miR-155-5p is one of the most studied miRNAs in TB disease and known to be dysregulated by infection in different systems (39). We found that the dysregulated targets of miR-155-5p are associated with activation of various immune cell types. Disruption of these coordinated pathway cascades may impede the host’s ability to recognize and respond to *Mtb* infection (5, 6). Existing literature shows that miR-155-5p is involved in regulating cellular immune responses to *Mtb*, but that it may have both host-beneficial (i.e., increased survival of *Mtb*-specific T cells) and host-detrimental (i.e., suppression of autophagy) effects depending on when it is expressed and what genes are targeted (40–42).

While miR-125b-5p was not significantly dysregulated in our study, it has been shown to be upregulated by qRT-PCR in previous studies. Previously, miR-125b-5p was found to be upregulated in *Mtb* infection and upregulation was associated with lower levels of TNF, suggesting suppression of an inflammatory response (12). We found significantly altered expression of miR-125b-5p targets, which are involved in regulation of metabolism, lipid processing, and small molecular transport and subcellular localization. This may suggest that even small changes in miRNA expression that failed to meet our stringent statistical thresholds may still have biological impact. Also, given that gene targets within any pathway can be targeted by multiple miRNAs, there may be redundancies in which more than one miRNA may target different genes within the same pathway, such that the expression change in each individual miRNA may be small, but the combined effects are sufficient to alter the activation of the downstream pathway. This is represented by functional enrichment pathways for miR-125b-5p, miR-27a-3p, and miR-22-3p. These three miRNAs all targeted pathways involved in regulation of metabolism and lipid processing, disruption of which may increase access to nutrients and facilitate *Mtb* persistence (30, 32, 43).

In line with the likely additive or complimentary effects of multiple non-significantly dysregulated miRNAs that target the same pathways, miR-27a-3p was also not significantly dysregulated, though its dysregulated targets were involved in biosynthetic and metabolic processes, as described above. miR-27a-3p has been shown previously to be involved in increasing production IFN*γ*, IL-1β, IL-6, and TNFα, which are key cytokines associated with a successful anti-TB response (44) and inhibition of intracellular survival of non-tuberculous mycobacteria (45).

While miR-22-3p is less well characterized in TB models, we found it to be significantly downregulated in *Mtb*-infected primary MDMs by sRNA-seq. Though this is the first time it has been described in macrophages, miR-22-3p has been found to be differentially abundant in the plasma of TB patients vs. healthy controls and has been considered for inclusion as a potential blood-based TB biomarker (46). More work should be done to investigate the role of miR-22-3p in *Mtb* infection.

Both miR-30b-5p and miR-30c-5p were significantly downregulated in our study. Like miR-155-5p, the miR-30 family has been implicated in host immunity to TB, given its known role in targeting genes involved in important anti-TB responses, such as autophagy (47). While the GO : BP enrichment analysis of differentially expressed miR-30c-5p targets showed significant involvement in exocytosis and secretion, which are important for cell-to-cell signaling, the miR-30b-5p and miR-30c-5p target, IL-1α, was significantly upregulated and is known to induce autophagy in macrophages (48, 49). Taken together, these findings suggest that downregulation of miR-30c-5p and miR-30b-5p in *Mtb* infection may reflect the macrophage’s effort to induce autophagy and cell-to-cell communication *via* exocytosis.

miR-29a-3p was included in our studies, as it has been found previously to be dysregulated in human MDMs infected with *Mycobacterium avium* by qPCR and was shown to target caspase 7, which is involved in apoptosis (50). While we did not observe significant dysregulation of miR-29a-3p in *Mtb*-infected primary human MDMs by sRNA-seq, targets of miR-29a-3p, including those involved in angiogenesis, which is a critical process involved in the formation of granulomas and nutrient acquisition in *Mtb* infection (31, 51, 52), were significantly differentially regulated. The lack of dysregulation of miR-29a-3p expression in our study may suggest that dysregulation of angiogenesis may be mediated *via* altered macrophage responses, but occur independently of miR-29a-3p. This may be an important relationship to pursue in more complex animal models in which blood vessel formation may influence granuloma formation and dissemination of disease.

We found dysregulation of miR-191-3p, which has not yet been well-characterized in TB disease, though there were only two differentially expressed target genes, which did not associate with a specific biological process. Nonetheless, miR-191-3p has been implicated in other disease states and may warrant further study (53).

Unlike previous studies, we found that miR-21-5p was significantly downregulated in *Mtb*-infected MDMs. Earlier studies have shown upregulation of miR-21-5p in *Mtb*-infected murine RAW264.7 cells and human THP1 cells, which are both cancer-derived macrophage cell lines. Induction of miR-21-5p expression was associated with increased *Mtb* survival and reduced production of inflammatory cytokines, such as IL-1β, IL-6, and TNFα (54). Conversely, we found that miR-21-5p was downregulated while its target, IL-1β, was significantly upregulated. This suggests that compared to the cancerous cell lines, primary MDMs may generate more pro-inflammatory cytokines through downregulation of miR-21-5p.

We included miR-223-5p in our analyses based on previous literature, which has shown increased susceptibility of miR-223-5p knock out mice to TB (10). In our study, we did not find miR-223-5p to be significantly dysregulated or a significant pathway association for the differentially expressed miR-223-5p target genes. This could indicate differences in the roles of this miRNA between model systems, such that changes in miR-223-5p expression are more important in non-macrophage cell types that are present *in vivo*. For instance, increased susceptibility of miR-223-5p knock out mice to TB disease is associated with robust neutrophil-mediated lung inflammation, which suggests that neutrophils may be critical for mediating the effects of miR-223-5p expression changes (10). It may also indicate that changes in miR-223-5p expression must be quite large in order to have a biological effect.

Analysis of genome wide methylation changes in *Mtb*-infected cells by WGBS showed no significant methylation of promoter or gene regions of candidate miRNAs, suggesting that changes in the expression of miRNAs is likely to occur in a methylation-independent manner. On the other hand, various differentially expressed genes were also found to be differentially methylated by WGBS following *Mtb* infection in macrophages. Like differentially expressed genes targeted by the 10 miRNA candidates, macrophage genes that were both differentially expressed and divergently methylated following *Mtb* infection were involved in various pathways important for anti-*Mtb* responses. Importantly, hypermethylated genes appeared to be involved in driving activation of innate and adaptive immune cells. This suggests that one mechanism by which *Mtb* may suppress immune activation is by increasing methylation of genes involved in related pathways in infected macrophages. Alternatively, hypomethylated genes were most significantly associated with the positive regulation of metabolic processes, which may underlie the ability of *Mtb* to alter macrophage metabolic processes to increase access to nutrient sources and promote bacterial growth and survival.

Finally, integration of sRNA-seq and RNA-seq data with WGBS showed that processes affected by changes in miRNA and mRNA expression are also divergently methylated. Most DMRs were hypomethylated, which may indicate the macrophage’s effort to open chromatin to allow for rapid changes in transcriptional reprogramming during infection. Interestingly, in line with our miRNA and mRNA data, hypermethylation was associated with suppression of immune cell activation, while hypomethylation was associated with enhanced macrophage metabolism. Together, these opposing effects may reflect the generation of an environment in which intracellular bacilli are shielded from immune-mediated killing and able to access metabolic resources required for growth and survival within the host.

Genes that were differentially expressed, divergently methylated, and targeted by at least one miRNA of interest were significantly associated with the AMPK signaling pathway, which is central to various cellular processes that shape the response to *Mtb* infection, including regulation of autophagy, fatty acid biosynthesis, glucose metabolism, and cell proliferation (55). This pathway is of particular importance given that AMPK-targeting host-directed therapies, such as metformin, have been shown to promote *Mtb* killing in macrophages and in lungs of *Mtb*-infected mice (56) and to improve mortality when included in treatment regimens for TB patients with diabetes mellitus (57).

Our data, combined with findings from previous literature, suggest that miRNAs are multifunctional in that they may target multiple genes from redundant and complimentary pathways. Additionally, multiple miRNAs may co-regulate the same targets or pathways, which indicates that even small changes in a group of miRNAs may be biologically relevant. Additionally, alternate forms of gene regulation, including DNA methylation changes, also control miRNA-targeted pathways during *Mtb* infection of macrophages. This emphasizes the complexity of regulation of host responses and shows that the overall response of the macrophage is shaped by multiple contributing factors that must coordinate with one another in order to generate the appropriate response required to eliminate infection. While reductionist evaluations of these regulatory elements are powerful for determining specific targets, to understand how they shape disease outcome, they must also be While reductionist evaluations of these regulatory elements are powerful for determining specific targets, to understand how they shape disease outcome, they must also be studied in the context of broader regulatory networks.

These large scale transcriptomic and methylomic studies are descriptive by nature. This study is not intended to investigate the impact of specific dysregulated elements on overall outcome of *Mtb* infection, but rather to provide an unbiased, global analysis of the complex network of macrophage transcriptional regulation, and to provide a road map for future studies exploring the relationship between pre-transcriptional DNA methylation and post-transcriptional miRNA regulatory mechanisms. Additional work should focus on targeted analysis of candidate miRNA-differentially expressed gene target pairs and genes which are both differentially methylated and differentially expressed. Additionally, further analysis of changes in miRNA expression and methylation, particularly related to the activation of the AMPK pathway, should be examined *in vivo*. Overall, these studies show that dysregulation of methylation and miRNA candidate target genes centers on suppression of immune activation. Understanding how to restore the balance of these elements may have implications for altering outcome of *Mtb* infection in macrophages, particularly in the context of developing host-directed therapies which target AMPK signaling and other key regulatory pathways.

## Data Availability Statement

The datasets presented in this study can be found in online repositories. The names of the repository/repositories and accession number(s) can be found below: https://www.ncbi.nlm.nih.gov/geo/, GSE164287 https://www.ncbi.nlm.nih.gov/geo/, GSE151050.

## Author Contributions

ML was responsible for the majority of the presented conceptualization, experimental work, data analysis, data visualization, and manuscript writing. RL assisted with key experimental work and editing, MKH and PCK were responsible for conception and design of the study, provision of necessary resources, and critical revision of the manuscript. All authors contributed to the article and approved the submitted version.

## Funding

This work was supported by National Institutes of Health (grant numbers UH3AI122309, K24AI143447). NIH grant number R01HL149450 (all grants to PCK).

## Conflict of Interest

The authors declare that the research was conducted in the absence of any commercial or financial relationships that could be construed as a potential conflict of interest.

## Supplementary Material

Supplementary Figure 1 Experimental workflow diagram. Platelet-depleted whole blood from healthy human donors was used for isolation of primary human monocytes. Peripheral blood mononuclear cells (PBMCs) were isolated using Ficoll-paque gradient separation. PBMCs were incubated in serum-free media for four hours to allow for monocyte adherence to cell culture plates. After incubation, non-adherent lymphocytes were washed away with 1X PBS. Monocytes were allowed to differentiate in complete media containing 10% FBS for 1 week, and then infected with luminescent Mtb (H37Rv-lux) for 24 or 48 hours. At each time point, cells were lysed in TRIzol for RNA extraction, library preparation, and sequencing. Sequencing analysis was performed using miRge2.0 and DESeq2 packages in R.

Supplementary Figure 2 Comparison of miRNA expression between 24 and 48 hours. Volcano plot of differentially expressed miRNAs in uninfected or infected MDMs 24 and 48 hours after infection of infected conditions. n = 4 independent human donors. Red lines represent significance thresholds. Red dots represent significantly differentially expressed miRNAs. Differentially expressed miRNAs are reported with their Log2(Fold Change) (Log2(FC)) and adjusted *p*-values.

Supplementary Table 1 Differentially expressed miRNAs from small RNA sequencing. See attached **Supplemental_Table_1_DEmiRs.csv file**.

Supplementary Table 2 Differentially expressed mRNAs from total RNA sequencing. See attached **Supplemental_Table_2_DEmiRs.csv file** file.

Supplementary Table 3 Functional enrichment analysis of differentially expressed mRNAs. See attached **Supplemental_Table_3_DEmiRs.csv file** file.

Supplementary Table 4 Contingency table for enrichment of dysregulated miRNA targets. Chi-square analysis with Yates correction to determine if dysregulated genes are statistically enriched for miRNA targets. Total number of candidate miRNA targets was determined by miRNet (38). Total number of genes was based on total genes detected by RNA-seq. Genes with low reads (sum across all samples less than 10 reads) were filtered out prior to analysis.

Supplementary Table 5 Differential methylation from whole genome bisulfite sequencing. See attached **Supplemental_Table_5_DEmiRs.csv file** file.

Supplementary Table 6 Contingency table for enrichment of AMPK pathway dysregulation. Chi-square analysis with Yates correction to determine if dysregulated genes (differentially methylated or expressed) are statistically enriched for AMPK pathway genes. Total number of AMPK Pathway Genes was based on KEGG pathway. Total number of genes was based on total genes detected by RNA-seq. Genes with low reads (sum across all samples less than 10 reads) were filtered out prior to analysis.
