# Supplementary figures and images for: Key Macrophage Responses to Infection With Mycobacterium tuberculosis Are Co-Regulated by microRNAs and DNA Methylation
Source: Front Immunol. 2021 Jun 1;12:685237. doi: 10.3389/fimmu.2021.685237 (PMC8204050; doi:10.3389/fimmu.2021.685237)

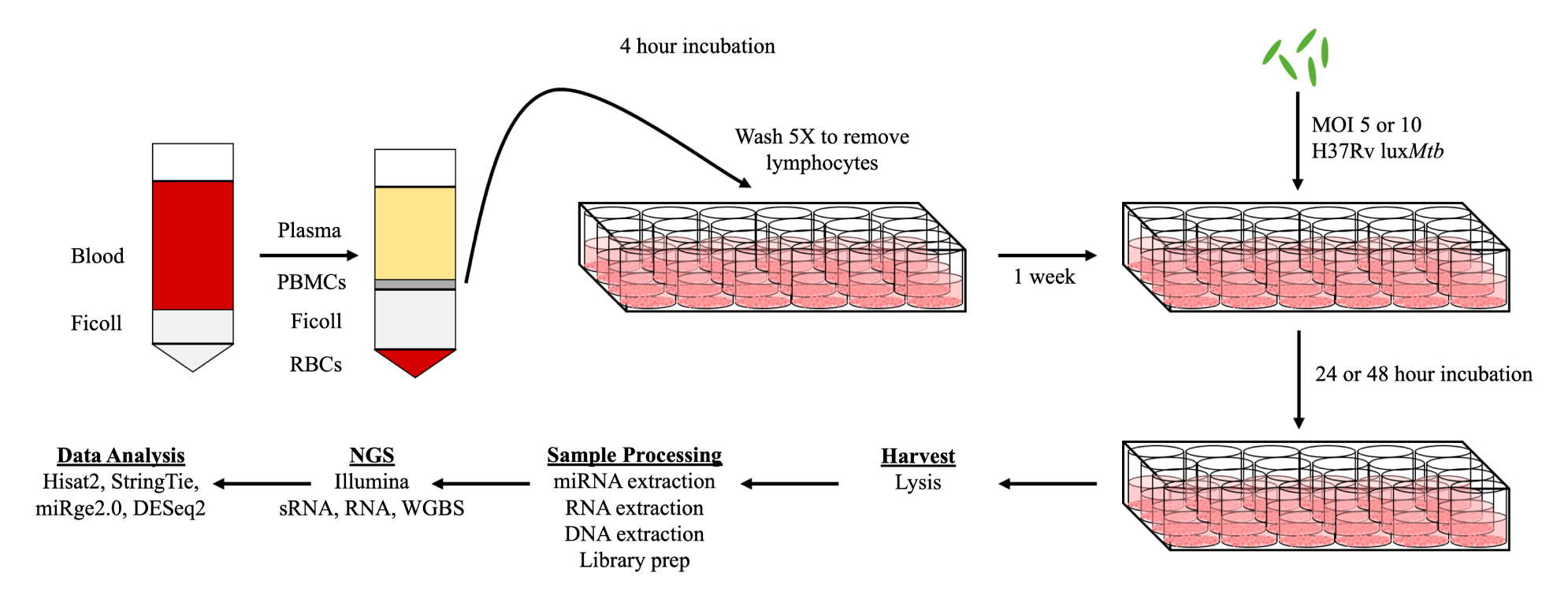

Supplement: Supplementary Figure 1 — Experimental workflow diagram. Platelet-depleted whole blood from healthy human donors was used for isolation of primary human monocytes. Peripheral blood mononuclear cells (PBMCs) were isolated using Ficoll-paque gradient separation. PBMCs were incubated in serum-free media for four hours to allow for monocyte adherence to cell culture plates. After incubation, non-adherent lymphocytes were washed away with 1X PBS. Monocytes were allowed to differentiate in complete media containing 10% FBS for 1 week, and then infected with luminescent Mtb (H37Rv-lux) for 24 or 48 hours. At each time point, cells were lysed in TRIzol for RNA extraction, library preparation, and sequencing. Sequencing analysis was performed using miRge2.0 and DESeq2 packages in R. [file Image_1.tif]

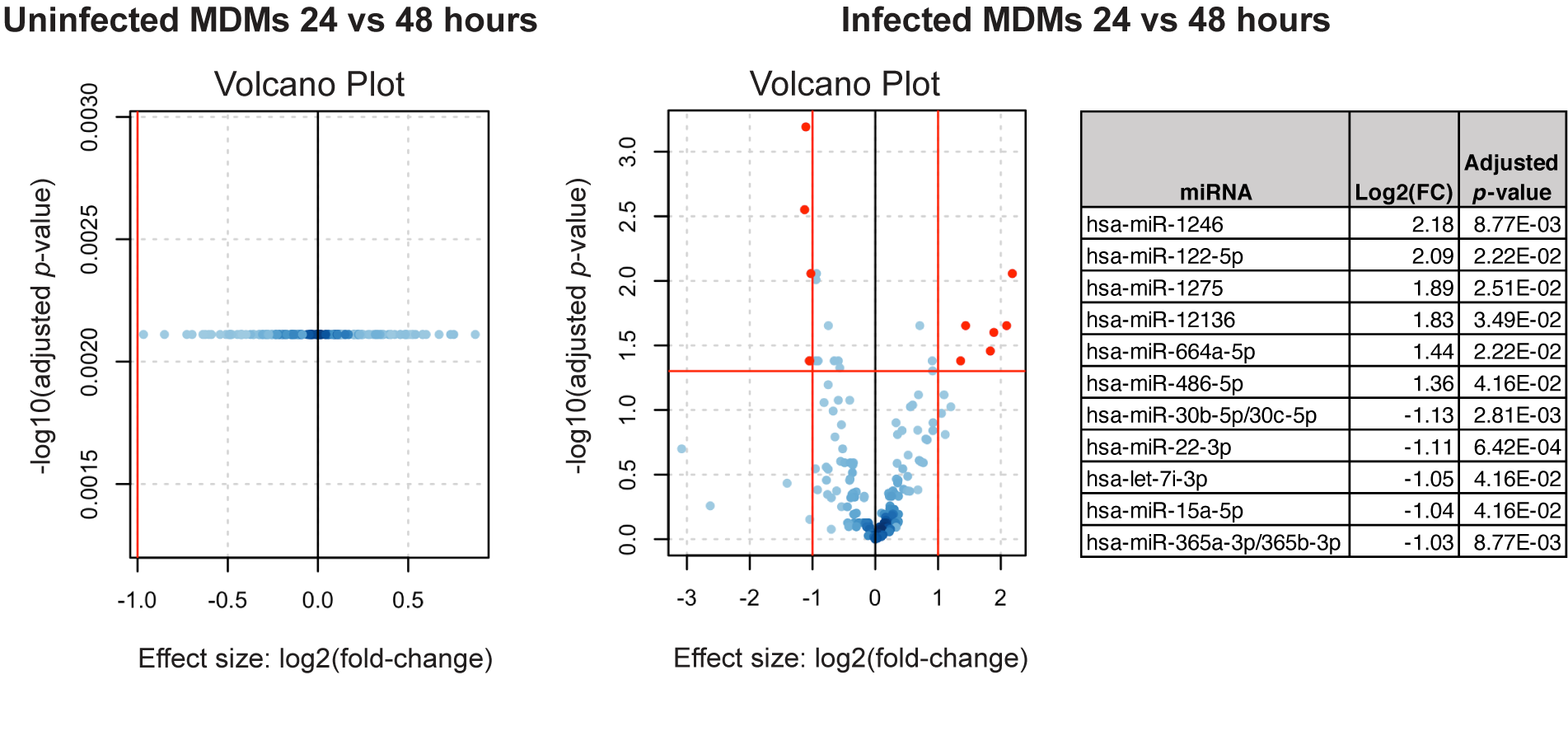

Supplement: Supplementary Figure 2 — Comparison of miRNA expression between 24 and 48 hours. Volcano plot of differentially expressed miRNAs in uninfected or infected MDMs 24 and 48 hours after infection of infected conditions. n = 4 independent human donors. Red lines represent significance thresholds. Red dots represent significantly differentially expressed miRNAs. Differentially expressed miRNAs are reported with their Log2(Fold Change) (Log2(FC)) and adjusted p-values. [file Image_2.tif]
