# Supplementary material for: Key Macrophage Responses to Infection With Mycobacterium tuberculosis Are Co-Regulated by microRNAs and DNA Methylation
Source: Front Immunol. 2021 Jun 1;12:685237. doi: 10.3389/fimmu.2021.685237 (PMC8204050; doi:10.3389/fimmu.2021.685237)
Supplement: Supplementary Table 4 — Contingency table for enrichment of dysregulated miRNA targets. Chi-square analysis with Yates correction to determine if dysregulated genes are statistically enriched for miRNA targets. Total number of candidate miRNA targets was determined by miRNet (38). Total number of genes was based on total genes detected by RNA-seq. Genes with low reads (sum across all samples less than 10 reads) were filtered out prior to analysis. [file Table_4.docx]

**Supplemental Table 4. Contingency table for enrichment of dysregulated miRNA targets.**

|  | Non-miRNA Targets | miRNA Targets | Total |
| --- | --- | --- | --- |
| Not dysregulated | 17083 | 2886 | 19967 |
| Dysregulated | 657 | 158 | 815 |
| **Total** | 17740 | 3044 | 20784 |
|  |  |  |  |
| Metric | X^2^ | z-score | *p*-value |
| Results | 14.86 | 3.86 | 0.0001 |

Chi-square analysis with Yates correction to determine if dysregulated genes are statistically enriched for miRNA targets. Total number of candidate miRNA targets was determined by miRNet ([38](#_ENREF_38)). Total number of genes was based on total genes detected by RNA-seq. Genes with low reads (sum across all samples less than 10 reads) were filtered out prior to analysis.
