# Supplementary material for: Key Macrophage Responses to Infection With Mycobacterium tuberculosis Are Co-Regulated by microRNAs and DNA Methylation
Source: Front Immunol. 2021 Jun 1;12:685237. doi: 10.3389/fimmu.2021.685237 (PMC8204050; doi:10.3389/fimmu.2021.685237)
Supplement: Supplementary Table 6 — Contingency table for enrichment of AMPK pathway dysregulation. Chi-square analysis with Yates correction to determine if dysregulated genes (differentially methylated or expressed) are statistically enriched for AMPK pathway genes. Total number of AMPK Pathway Genes was based on KEGG pathway. Total number of genes was based on total genes detected by RNA-seq. Genes with low reads (sum across all samples less than 10 reads) were filtered out prior to analysis. [file Table_6.docx]

**Supplemental Table 6. Contingency table for enrichment of AMPK pathway dysregulation.**

|  | Non-AMPK Genes | AMPK Pathway Genes | Total |
| --- | --- | --- | --- |
| Not dysregulated | 17934 | 45 | 17979 |
| Dysregulated | 2782 | 23 | 2805 |
| **Total** | 20693 | 68 | 20784 |
|  |  |  |  |
| Metric | X^2^ | z-score | *p*-value |
| Results | 22.43 | 4.74 | <0.0001 |

Chi-square analysis with Yates correction to determine if dysregulated genes (differentially methylated or expressed) are statistically enriched for AMPK pathway genes. Total number of AMPK Pathway Genes was based on KEGG pathway. Total number of genes was based on total genes detected by RNA-seq. Genes with low reads (sum across all samples less than 10 reads) were filtered out prior to analysis.
